# ‘Having the mask on didn't worry me until … they clamped my head down so I wouldn't move’: A qualitative study exploring anxiety in patients with head and neck cancer during radiation therapy

**DOI:** 10.1002/jmrs.658

**Published:** 2023-02-01

**Authors:** Erin Forbes, Kerrie Clover, Amanda L. Baker, Ben Britton, Melissa Carlson, Kristen McCarter

**Affiliations:** ^1^ School of Medicine and Public Health, College of Health Medicine and Wellbeing University of Newcastle Callaghan New South Wales Australia; ^2^ PsychoOncology Service, Department of Consultation Liaison Psychiatry Calvary Mater Newcastle Callaghan New South Wales Australia; ^3^ Hunter New England Mental Health Services Newcastle New South Wales Australia; ^4^ School of Psychological Sciences, College of Science, Engineering and Environment University of Newcastle Callaghan New South Wales Australia

**Keywords:** clinical site, claustrophobia, discipline, head and neck, general, general, psychology, general, research – qualitative, radiation oncology

## Abstract

**Introduction:**

More than 20% of patients undergoing radiation therapy for head and neck cancer report anxiety specifically related to the immobilisation mask, a tight‐fighting mask patients are required to wear for the duration of each treatment session. However, limited research has investigated this from the patient perspective. The aim of this study was to better understand patient experiences of mask anxiety during head and neck cancer radiation therapy and to explore patient attitudes toward potential strategies that may reduce mask anxiety during this treatment.

**Methods:**

Five patients with head and neck cancer, who had self‐reported mask anxiety during radiation therapy, participated in semi‐structured, qualitative interviews exploring their experiences of anxiety and suggestions for reducing anxiety. A codebook thematic analysis was conducted.

**Results:**

Six main themes were identified: (1) triggers of anxiety; (2) adjusting to radiation therapy; (3) education about the mask; (4) coping; (5) motivation and (6) improving the patient experience.

**Conclusion:**

Findings from these interviews provide valuable insight into how and when healthcare providers may be able to assist patients to manage mask anxiety. Recommendations include increased communication from health care providers; delivery of visual information to improve patient preparedness; exposure/opportunities to interact with the masks prior to treatment commencing and increased control of music/soundtrack selection. However, a limitation of this study is the small sample size and further research is warranted.

## Graphical abstract

Patients undergoing radiation therapy for head and neck cancer experience anxiety specifically related to the immobilisation mask, a tight‐fighting mask patients are required to wear for the duration of each treatment session. The findings from this research provides valuable insight into how and when healthcare providers may be able assist patients to manage mask anxiety. Recommendations include increased communication from health care providers; delivery of visual information to improve patient preparedness; exposure/opportunities to interact with the masks prior to treatment commencing and increased control of music/soundtrack selection.

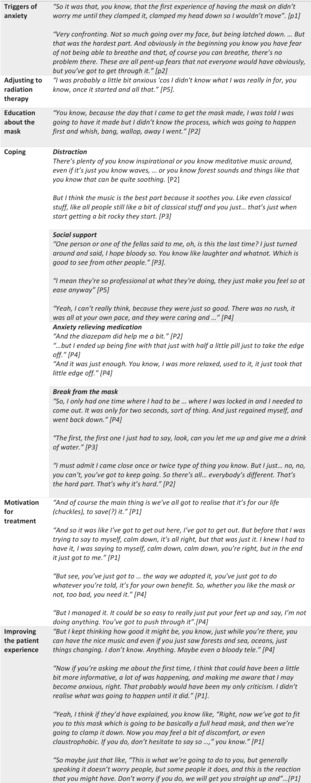

## Introduction

Between 10 and 20% of all patients with cancer experience significant symptoms of anxiety prior to radiation therapy.^1^ Radiation therapy for head and neck cancer (HNC) sees patients experience anxiety at an even higher rate, with up to 26% of patients reporting moderate or severe distress.^2^, ^3^ One of the reasons for this increased anxiety is the use of an immobilisation mask, a tight‐fitting mask that patients are required to wear for the duration for each treatment session. The immobilisation mask is made from thermoplastic perforated mesh which has been softened in a warm water bath and moulded around the patient's head, sometimes including the neck and shoulders and secured to the treatment couch during treatment.^4^

The use of the immobilisation mask is standard care to ensure safe and accurate delivery of the radiation dose, by limiting the patient's movement.^5^ However, the restrictive nature of the mask can heighten anxiety for many patients^2^, ^3^, ^6^ and wearing the mask has been described as ‘feeling like being buried alive’.^7^ Although anxiety reduces over time for most patients, there remain subsets of patients for whom anxiety persists (22%) or increases throughout the treatment course (6%).^8^ Strategies to assist patients to cope with the mask vary between cancer treatment centres and include making changes to the mask (cutting out the eyes and mouth to reduce the restrictive feelings), offering music in the treatment bunkers and administration of pharmacological anxiolytics (such a benzodiazepines).^9^ However, there are no clear guidelines or existing evidence to guide clinicians on how and when to provide support to patients who may be experiencing significant mask anxiety.

Nixon et al^3^ explored the experience of anxiety in (Australian) patients (*n* = 20) undergoing radiation therapy for head and neck cancer and identified two key themes: contributors to mask anxiety and managing mask anxiety during treatment. Participants reported that vulnerabilities to claustrophobia (pre‐existing claustrophobia), their physiological or psychological responses to the experience and their expectations of the treatment were all contributors to their experience of mask anxiety. Participants reported using a number of different strategies to manage their anxiety (including cognitive strategies, pharmacological strategies and practical strategies), and a number of different mindsets (e.g., self‐determination or feeling that there is no other choice). A similar study conducted by Keast and colleagues^10^ of HNC survivors (*n* = 20), identified seven themes: information received by participants; potential predictors of mask anxiety; reactions to the mask; trajectories of mask anxiety; supportive behaviour and communication of health professionals; coping with the mask and thoughts and feelings about the mask. While these papers provide robust and important contributions to understanding the experience of mask anxiety in radiation therapy, they do not consider how the patient experience could be improved. The current paper aims to build on these findings by exploring patient views/insights of how patient care could be improved with regard to mask‐related anxiety during treatment sessions. This study will explore: (1) the patient experience of mask anxiety during radiation therapy and (2) patient attitudes toward potential strategies that may reduce mask anxiety during treatment.

## Methods

The study is reported in accordance with the Consolidated criteria for Reporting Qualitative research (COREQ) checklist^11^ (see Appendix S1). A thematic analysis, with an inductive approach was used for this study.^12^ This study was nested within a larger longitudinal observational study, and therefore descriptive quantitative data, collected in the larger study, are also reported.

### Sample

A purposive sampling strategy was employed for this study, with a target sample of *n* = 8. In line with thematic analysis, the sample size was chosen based on the study team's previous experience conducting qualitative studies with similar patient groups. It was expected that this sample size would be feasible to recruit, considering the small recruitment pool (18 eligible participants) and the vulnerability of the cohort, while still providing sufficient breadth and depth of data. Given the narrow aims of our study, and the specific characteristics of our sample, the authors agreed that this sample size would provide sufficient information power.^13^

The study team asked participants who had been recruited to a larger study (not yet published) if they would be interested in participating in an interview exploring the experience of radiation therapy for patients with HNC. The larger study was a longitudinal observational study which aimed to validate a screening tool for early identification of patients with HNC who are likely to experience anxiety during treatment. In this study, self‐reported anxiety data were collected from participants prior to the commencement of treatment, on the day of treatment planning, and at the first, second, third and twentieth treatment sessions. Clinical data was also extracted from the medical record.

Of the 101 participants in the parent study (adult patients diagnosed with HNC requiring a mask for radiation therapy), a subset of 18 eligible participants were identified. Participants were eligible if they were identified as having experienced anxiety during their treatment on any one of the four following items: (1) required a break from the mask; (2) required anxiolytic medication; (3) reported ≥4 on the anxiety thermometer^14^; or (4) reported ≥4 on the distress thermometer^15^, ^16^ in the larger study.

Eligible participants were initially contacted via mail with an invitation to participate in a qualitative interview. Participants were sent an information and consent form, together with a reply‐paid envelope (to return the completed consent form and indicate their interest). The study team followed up with a phone call to offer the opportunity to ask any questions they had about the study. For those who confirmed an interest in the study, an interview was arranged either face‐to‐face at the Calvary Mater Hospital (Newcastle, Australia), or over the phone, as per the participant's preference.

### Qualitative data collection

A semi‐structured interview protocol was used to guide the interviews with participants. The interviews explored the experience of radiation therapy generally, feelings about the immobilisation mask and strategies that may help to reduce anxiety. These questions were selected based on the research team's understanding of the patient experience in radiation therapy and their clinical experience. Several members of the research team (EF, KC, BB) have experience working with patients undergoing radiation therapy for HNC requiring an immobilisation mask. EF in a research role, and KC and BB in a clinical capacity. Our experience and expertise were instrumental in the development of our interview guide for this research.

Interviews took place between February 2019 and February 2020. They were conducted by the lead author (EF), a research assistant and PhD candidate, who had completed qualitative research training via an undergraduate course. EF was also supervised by other members of the research team with qualitative research experience. EF was known to two of the participants, from prior involvement in the larger study. However, we do not believe this relationship impacted the interview findings, as EF had no clinical involvement with the participants and had primarily interacted with them for data collection purposes.

Interviews were audio recorded by the interviewer and transcribed verbatim. All participants provided written or audio recorded verbal consent as per the consent protocol approved by Hunter New England Human Research Ethics Committee. The researchers mailed a copy of the transcripts to each participant to allow participants an opportunity to comment or make any corrections and ensure that the findings were being presented in an accurate manner.

### Quantitative measures

#### Distress thermometer

The distress thermometer was used as a measure of mask anxiety in the larger study. The distress thermometer is a single item self‐report, continuous 11‐point scale, with 0 indicating no distress and 10 indicating extreme distress. It is well established that a score of **≥**4 indicates clinically significant distress.^15^, ^16^

#### Anxiety thermometer

Like the distress thermometer, the anxiety thermometer was also used as a measure of mask anxiety in the larger study. The anxiety thermometer is a single item continuous 11‐point scale, with 0 indicating ‘not at all anxious’ and 10 indicating ‘extremely anxious’. Consistent with the distress thermometer, a cut off score of **≥**4 was used to indicate significant anxiety.^14^

### Data analysis

#### Qualitative data

Data were analysed using codebook thematic analysis.^17^ Initially, two coders (EF and MC) listened to the audio recorded interviews to familiarise themselves with the data. The coders then independently free coded two transcripts (independent parallel coding) with NVivo 12 qualitative data analysis software. Following discussion, this coding formed the basis of a preliminary codebook. The coders then continued to code transcripts, discuss and refine the codebook. Following this, EF clustered the codes to identify patterns in the data, reviewed the preliminary patterns and then named the themes.^18^ The themes were then discussed among three members of the research team (KC, AB and KM) and the theme names were amended and finalised.

#### Quantitative data

Participant demographics and previously collected observational data were analysed using IBM SPSS Statistics for Mac version 28.

## Results

Of the 18 participants contacted, five provided consent to participate, nine declined to participate, one was deceased and three were not able to be contacted. One participant had a support person present for the duration of the interview. Four interviews were completed face to face (at the Calvary Mater Hospital) and one was completed over the phone. Interviews ranged between 13 min and 43 min (with a mean of 25 min). The sample was all male with a mean age of 65 years (SD = 7.24, range 54–74). Additional demographic and clinical characteristics of the sample are detailed in Table 1.

**Table 1:** Patient demographics and characteristics.

Sociodemographics	Total sample (*n* = 5)	
	M (SD)	Range
Age (years)	65.36 (*7.24*)	54.30–74.23
*Marital status*		
Married	3 (60%)	
Defacto	1 (20%)	
Single	1 (20%)	
*Lives alone*		
No	4 (80%)	
Yes	1 (20%)	
*Mental health history*		
Claustrophobia	1 (20%)	
Depression	1 (20%)	
*Chemotherapy*		
No	4 (80%)	
Yes (concurrent)	1 (20%)	
*Cancer site*		
Cutaneous	4 (80%)	
Other	1 (20%)	
*Stage*		
I	1 (20%)	
II	2 (40%)	
III	1 (20%)	
IV	1 (20%)	
*Radiation therapy intent*		
Curative	4 (80%)	
Palliative	1 (20%)	
Time since radiation therapy	625 days (1 year and 8 months)	133–1087 days (4 months–2 years and 11 months
Break required from mask	2 (40%)	
Anxiolytic medication required	3 (60%)	
Referred to occupational therapist for relaxation	2 (40%)	
**≥**4 Distress Thermometer	3 (60%)	
**≥**4 Anxiety Thermometer	5 (100%)	

The value in italic is the standard deviation.

## Themes

Six main themes were identified from the data relating to the patient experience of radiation therapy: (1) triggers of anxiety; (2) adjusting to radiation therapy; (3) education about the mask; (4) coping; (5) motivation and (6) improving the patient experience. Exemplary verbatim quotes are provided to illustrate the themes appropriately (Table 2), with numbers provided for each participant to protect confidentiality.

**Table 2:** Exemplary quotes.

Triggers of anxiety	“So it was that, you know, that the first experience of having the mask on did not worry me until they clamped it, clamped my head down so I would not move”. [P1] “Very confronting. Not so much going over my face, but being latched down. … But that was the hardest part. And obviously in the beginning you know you have fear of not being able to breathe and that, of course you can breathe, there's no problem there. These are all pent‐up fears that not everyone would have obviously, but you have got to get through it.” [P2]
Adjusting to radiation therapy	“I was probably a little bit anxious ‘cos I did not know what I was really in for, you know, once it started and all that.” [P5].
Education about the mask	“You know, because the day that I came to get the mask made, I was told I was going to have it made but I did not know the process, which was going to happen first and whish, bang, wallop, away I went.” [P2]
Coping	*Distraction* There's plenty of you know inspirational or you know meditative music around, even if it's just you know waves, … or you know forest sounds and things like that you know that can be quite soothing. [P2] But I think the music is the best part because it soothes you. Like even classical stuff, like all people still like a bit of classical stuff and you just… that's just when start getting a bit rocky they start. [P3]
	*Social support* “One person or one of the fellas said to me, oh, is this the last time? I just turned around and said, I hope bloody so. You know like laughter and whatnot. Which is good to see from other people.” [P3]. “I mean they are so professional at what they are doing, they just make you feel so at ease anyway” [P5] “Yeah, I cannot really think, because they were just so good. There was no rush, it was all at your own pace, and they were caring and …” [P4]
	*Anxiety relieving medication* “And the diazepam did help me a bit.” [P2] “…but I ended up being fine with that just with half a little pill just to take the edge off.” [P4] “And it was just enough. You know, I was more relaxed, used to it, it just took that little edge off.” [P4]
	*Break from the mask* “So, I only had one time where I had to be … where I was locked in and I needed to come out. It was only for two seconds, sort of thing. And just regained myself, and went back down.” [P4] “The first, the first one I just had to say, look, can you let me up and give me a drink of water.” [P3] “I must admit I came close once or twice type of thing you know. But I just… no, no, you cannot, you have got to keep going. So there's all… everybody's different. That's the hard part. That's why it's hard.” [P2]
Motivation for treatment	“And of course the main thing is we have all got to realise that it's for our life (chuckles), to save(?) it.” [P1] “And so it was like I've got to get out here, I've got to get out. But before that I was trying to say to myself, calm down, it's all right, but that was just it. I knew I had to have it, I was saying to myself, calm down, calm down, you are right, but in the end it just got to me.” [P1] “But see, you have just got to … the way we adopted it, you have just got to do whatever you are told, it's for your own benefit. So, whether you like the mask or not, too bad, you need it.” [P4] “But I managed it. It could be so easy to really just put your feet up and say, I'm not doing anything. You've got to push through it”.[P4]
Improving the patient experience	“But I kept thinking how good it might be, you know, just while you are there, you can have the nice music and even if you just saw forests and sea, oceans, just things changing. I do not know. Anything. Maybe even a bloody tele.” [P4] “Now if you are asking me about the first time, I think that could have been a little bit more informative, a lot of was happening, and making me aware that I may become anxious, right. That probably would have been my only criticism. I did not realise what was going to happen until it did.” [P1]. “Yeah, I think if they'd have explained, you know like, “Right, now we have got to fit you to this mask which is going to be basically a full head mask, and then we are going to clamp it down. Now you may feel a bit of discomfort, or even claustrophobic. If you do, do not hesitate to say so …,” you know.” [P1] “So maybe just that like, “This is what we are going to do to you, but generally speaking it does not worry people, but some people it does, and this is the reaction that you might have. Do not worry if you do, we will get you straight up and”…[P1]

### Theme 1: Triggers of Anxiety

Participants cited three triggers of anxiety including treatment anticipation (*n* = 2), discomfort (*n* = 2) and related claustrophobia (*n* = 5).

#### Anticipation

The anticipation of coming into the radiation therapy department to receive the treatment was considered a time of heightened anxiety

I think now that you've got to come over and have it done. Because I think it's always in the back of your mind while you're walking to the radiation… You're knowing that, oh you know I've got to go… [P3]

#### Discomfort

Participants indicated elements that caused discomfort such as lying flat on their back, bright lights in the treatment room and the inability to swallow comfortably (as a side effect of radiation) all contributed to their feeling of anxiety about the treatment.

Later on, in the … as it progressed, I had times where I wasn't able to swallow, and that would frighten me a little bit. [P4]

#### Claustrophobia

Participants reported claustrophobia as the primary precipitant of anxiety. In particular, most participants referred to the moment the mask was “clamped” [P1] or “latched down” [P2].

### Theme 2: Adjusting to radiation therapy

Participants cited that anxiety reduced in intensity after a period of adjustment to radiation therapy, and that when they were familiar with the treatment procedures, it became routine. Knowing “what to expect” [P1] and being aware of “what I had to come” (P1] were cited as key mediators of anxiety.

### Theme 3: Education about the mask

Participants indicated that they did not feel adequately prepared or informed before commencing radiation therapy. Several participants expressed a desire for more, or clearer information than they had received.

Visual information was cited as a useful tool in preparing for radiation therapy. One participant had watched a YouTube video prior to treatment (sent by a relative, not the healthcare team)

Well, I saw a video that was sent to me by my sister actually I think it was, on YouTube. Now if that had been shown (by the healthcare providers) it would have been a great education to see what was going to happen with me… [P2]

While most participants reported a desire for more information, some participants were apprehensive about the ability of staff to provide any further information prior to treatment, acknowledging how varied treatments must be between patients

Because, well as you know all the treatments are all different. So you virtually can't just say, well this is what we're going to put on you and it's not the one they're going to use (referring to the mask) because… I've seen people in there with full face masks on, come down to their shoulders and whatnot. You know, the top body. [P3]

### Theme 4: Coping strategies

Various coping strategies were employed by the participants to manage their anxiety during the treatment sessions, including distraction, social support, anxiety‐relieving medication and taking a break from the mask.

#### Distraction

Music was cited by most, as a valued coping tool for “taking your mind away from it” [P3]. Participants reported using music for relaxation and keeping track of the passage of time. Music is routinely played in the department where study participants were treated, however, the choice of music was considered important in mitigating anxiety, with one participant reporting that “I wouldn't have ZZ Top playing anyway. I'd prefer just something nice and quite relaxing” [P5]. Many participants reported a desire for relaxing music and one participant suggested some relaxing sounds like “forest sounds” [P2].

One participant also reported using the songs to gauge how long they had been in treatment and how long they had remaining

Well, I was drumming away once I got more relaxed to it, on my tummy, just to the beat and I knew the songs were getting through, you could judge that length of song to my treatment and know, oh it's nearly done. [P4]

Participants reported other forms of distraction had been helpful

But I used to look forward to the particular one where it came over (the treatment machine) and the little smiley or whatever it was (a sticker on the machine). I think I got a photo of it. And it just made me think of my grandkids. I associated them when that came. And that was sort of halfway, three‐quarters of the way through. [P4]

#### Social support

All participants reported gaining comfort from social support. Social supports varied from person to person, with some receiving support from other patients in the waiting room “one of the fellas said to me, “oh, is this the last time?” I just turned around and said, “I hope bloody so”. You know, like laughter and whatnot” [P3]. Others referred to support from a loved one who had accompanied them to hospital “also the fact that I had a calming influence of [wife]” [P1]. Participants often reported support from family/loved ones in general terms, for example, referring to support their loved one provided with practical help at home “So, they were there looking after the house and cooking and the other things” [P4]. However, the support provided by clinicians was generally more specific to the experience of anxiety “I mean they're so professional at what they're doing, they just make you feel so at ease anyway” [P4].

#### Anxiety‐relieving medication

Three of the participants had used anxiolytic medication during some of the treatment sessions to cope with their anxiety. Participants described the medication (diazepam) as “taking the edge off” [P4] and “calmed me down” [P2]. One participant suggested that “anyone who has to go through it, it's worth not being too proud and taking those, because it just helped.” [P4].

#### Break from mask

Two participants required a break from the mask during treatment planning and/or treatment in order to continue, while another participant reported that they “came close” [P2].

### Theme 5: Motivation for treatment

Several patients noted the importance of the radiation therapy as a key motivation to continue with the treatment. Some clearly stated that the treatment is a means to save their life “it's for our life, to save it” [P1], while others were more ambiguous, but made comments to the same effect, stating “you need it” [P4], “it's for your own benefit” [P4] and “you've got to push through” [P4].

### Theme 6: Improving the patient experience

Participants were asked their thoughts about specific coping strategies proposed by the research team including a weighted blanket, mindfulness, an audio puzzle, aromatherapy and more information. One participant reported some more distraction would have been helpful, such as things to look at.

I kept thinking maybe either something that reflected off the roof, like nice scenery… Anything. Maybe even a bloody tele. [P4]

Another participant believed that different options might be effective for different people

… as we just said, there's many and varied people, and all of those things that are suggested, I would say if they help stuff it's terrific. [P1]

However, generally participants indicated that more information would be the most useful strategy to mitigate their anxiety. In particular, participants suggested that a more specific explanation of the mask fitting procedure would have helped them know what to expect, “Now if you're asking me about the first time, I think that could have been a little bit more informative” [P1]. One participant indicated that an informational video would have been helpful “Maybe if like, if you're one‐on‐one with a patient before they go, … show the procedures … say for instance I'm getting my mask fitted and you show a video of a person when they first go in to when they put the mask on, show them what's involved. That one, or one with the shoulders (referring to different types of masks) …” [P3]. Several participants also felt that a warning that they may feel anxious (and what would happen if they did become anxious), would have been appreciated.

… making me aware that I may become anxious, right… I didn't realise what was going to happen until it did. [P1]

## Discussion

In this qualitative study with patients who had experienced anxiety during radiation therapy for HNC, thematic analysis revealed new information regarding patient perceptions of how the treatment journey could be improved to minimise anxiety. We identified six key anxiety‐related themes: triggers of anxiety, adjusting to radiation therapy, education about the mask, coping, motivation for treatment and improving the patient experience.

Key triggers of anxiety included discomfort and the clamping of the mask to the treatment bed. While there may be little room to improve the comfort of the treatment bed (or side effects such as difficulty swallowing), adjustments that do not compromise treatment efficacy (e.g., dimming lights if possible, a leg roll under the knees for back comfort) may improve the experience for some patients. Similarly, clear communication prior to the mask being secured to the bed (for example, advising that the clips can be released in an emergency) may ease anxiety for some patients.

Participants reported that there was a period of adjustment to radiation therapy, in which anxiety is heightened due to the unknown nature of the procedure. This is consistent with previous research by Nixon et al^8^ and Keast et al,^10^ both indicating that for most patients, anxiety reduces as patients become more familiar with the mask.^8^ This finding was unsurprising and suggests that intervention is required prior to treatment, or very early in the treatment journey, to support participants during the adjustment period.

Consistent with previous research, participants generally felt unprepared and felt the information they received about the mask was inadequate.^10^ In line with this, patients may benefit from visual information demonstrating the mask fitting procedure (e.g., YouTube videos, as suggested by two participants). This may benefit patients in two ways; an opportunity to see and become familiar with the procedure, but also an opportunity to self‐identify anticipatory anxiety and notify the treating team. Some patients may also benefit from an opportunity to see/feel the mask, prior to the mask fitting (if anxiety is disclosed prior to fitting), or prior to treatment, as exposure to the mask may facilitate habituation and reduced anxiety. While this may not be feasible within radiation therapy appointments, allied health (e.g., occupational therapists), may be able to fulfil this need. Participants also expressed a desire for staff to provide a warning that the procedure may make some people feel anxious, and how to respond/notify the staff if they did. This is a particularly useful finding, as it is a simple adjustment to clinical practice that may improve the experience for many patients.

Participants reported several different coping strategies, some were internal, self‐directed coping strategies, that were unprompted by staff (e.g., thinking about grandkids, gauging treatment progression from song duration), and some were provided by the treating team (music, anxiety relieving medication, support). Although coping strategies were mostly consistent with the findings of Keast and colleagues,^10^ in contrast to Keast and colleagues, no participants reported that they received support by a psychologist in the present study. This may be due to differences in cancer centres, where anxiety may initially be managed by alternative healthcare providers (such as occupational therapists, as is the case in the cancer centre associated with the present study). Of the strategies that were facilitated by the treating team, there are improvements that can be made to clinical practice. Participants emphasised the importance of patient selection of music, to ensure the effect was calming. This finding is consistent with previous research^19^ and suggests that all patients should be given opportunity to self‐select the music (or soundtracks) that are audible during their treatment sessions. In line with findings by Keast and colleagues,^10^ support from staff was also cited by all participants as helpful. Participants spoke very highly of the support received by the clinical team when it came to managing their anxiety while wearing the mask (in particular, the radiation therapists). This finding suggests that future interventions targeting anxiety during treatment sessions may be best delivered by the radiation therapists, many of whom build a strong rapport with patients early in the treatment journey.

Participants stressed the importance of their treatment as a key motivator for continuing with treatment. This was consistent with previous qualitative investigations of this phenomenon.^3^, ^10^

### Limitations & future research

A limitation of this research is the sample size. Only 18 participants from the larger study (*n* = 101) were eligible (identified as having experienced mask anxiety) to participate, and therefore the pool of potential participants was limited. Many of the eligible participants declined to be interviewed. Some cited other health concerns and too much going on, while others cited that they wanted their experience to be behind them, that they did not wish to revisit that time in their lives. It may be that this subgroup had different experiences or different coping strategies to those that participated, and it is not known whether this subgroup may have contributed a different perspective. While we did not reach our target sample size, recommended sample sizes for studies using thematic analysis have ranged from 6–16, depending on the population.^20^ We feel that findings from this paper are important and further studies are required to replicate and extend those findings in other settings and countries. Another potential limitation of this study is the short length of the interviews, which averaged at 25 min. However, prior to the interviews participants were informed that the interviews could take up to 60 min, to allow ample opportunity to share their perceptions of the treatment journey. The length of the interviews is also within the range of previous research on these phenomena.^3^

Another limitation is the relative homogeneity of the sample on demographic and clinical characteristics, and recruitment from a single cancer centre. In particular, the sample was entirely male. A largely male sample is expected in this population, with males accounting for more than 70% of patients with HNC.^21^ The other clinical and demographic factors also reflected the composition of the larger study sample; however the results of this study may not apply to the experiences of people with different characteristics. Finally, participants in this study were from 4 months to 2 years and 11 months post radiation therapy. It is possible that participants who had recently completed treatment and those who are nearing 3 years post treatment may have different recollections of the treatment experience.

For future research, qualitative exploration with a focus group could be a valuable addition to the literature, particularly with a view to generate discussion around areas for improvement. Focus groups are commonly used in developing new services and the interaction between participants can allow them to build on others' ideas.^22^ It would also be useful to include a sample with greater gender representativeness. It has been well established that anxiety is more common among female patients in oncology settings,^23^ and therefore it is possible that female patients' would bring additional perspectives. Finally, we would also suggest an exploration of healthcare providers' (including radiation therapists, radiation oncologists, psychologists and occupation therapists) perceptions of strategies to improve the patient experience, to further refine our recommendations for improved clinical care.

## Conclusion

These interviews provide valuable insights into how and when healthcare providers may be able assist patients to manage mask anxiety. While patients appreciated the support and professionalism of the treating team some recommendations for improvement include increased (prior) communication about the mask‐fitting procedure; warning patients they may feel anxious about the mask and what to do in response; delivery of visual information to improve patient preparedness; exposure/opportunities to interact with the masks prior to treatment commencing and increased opportunities for personalised distraction (e.g., self‐selection of music/soundtrack).

## Funding

This research was supported by University of Newcastle Research Higher Degree Student Support and a Hunter Cancer Research Alliance PhD top‐up.

## Conflicts of Interest

No conflict of interests to declare.

## Ethics Approval and Patient Consent

All participants provided written or audio recorded verbal consent as per the consent protocol approved by the Hunter New England Research Ethics Committee and Registered with the University of Newcastle Human Research Ethics Committee (16/02/17/4.07).

## Supporting information


**Appendix S1:** COREQ (COnsolidated criteria for REporting Qualitative research) Checklist.

## Data Availability

Deidentified data will be made available from the corresponding author upon reasonable request.
